# Combined application versus topical and intravenous application of tranexamic acid following primary total hip arthroplasty: a meta-analysis

**DOI:** 10.1186/s12891-017-1429-0

**Published:** 2017-02-21

**Authors:** Pei Zhang, Yuan Liang, Pengtao Chen, Yongchao Fang, Jinshan He, Jingcheng Wang

**Affiliations:** 1 Dalian Medical University, Dalian, Liaoning 116044 China; 2 Department of Orthopedics, Clinical Medical College of Yangzhou University, Subei People’s Hospital, Nantong West Road 98, Yangzhou, 225001 China; 3 Department of Orthopedics, Clinical Medical College of Yangzhou University, Subei People’s Hospital of Jiangsu Province, Yangzhou, China

## Abstract

**Background:**

The use of intravenous (IV) or topical tranexamic acid (TXA) in total hip arthroplasty has been proven to be effective and safe in total hip arthroplasty. However, which of these two administration routes is better has not been determined. The combined administration of TXA has been used in total knee arthroplasty with satisfactory results. We hypothesized that combined application of TXA may be the most effective way without increased rate of thrombotic events such as deep vein thrombosis (DVT) and pulmonary embolisms (PE) in patients subjected to primary total hip replacement (THA). A meta-analysis was conducted to compare the efficacy and safety of the combined use of tranexamic acid (TXA) relative to topical or intravenous (IV) use alone for treatment of primary THA. The outcomes included total blood loss, postoperative hemoglobin decline, transfusion rates, and the incidence rates of deep vein thrombosis (DVT) and pulmonary embolisms (PE).

**Methods:**

We searched electronic databases including PubMed, EMBASE, the Cochrane Library, Web of Science, the Chinese Biomedical Literature database, the CNKI database, and Wanfang Data until September 2016. The references of the included articles were also checked for additional potentially relevant studies. There were no language restrictions for the search. The data of the included studies were analyzed using RevMan 5.3 software.

**Results:**

Seven studies met the inclusion criteria, encompassing a total of 1762 patients. Our meta-analysis demonstrated that total blood loss, postoperative hemoglobin decline, and transfusion rates were significantly lower for patients that received the combined treatment compared to patients that received either topical or intravenous administration of TXA. No statistical differences were found in the incidence of deep venous thrombosis (DVT) or pulmonary embolism (PE).

**Conclusion:**

The group that received the combined treatment had lower total blood loss, postoperative hemoglobin decline, and transfusion rates without an increased rate of thrombotic events (DVT or PE). The topical or intravenous use of TXA in primary THA is generally considered to be safe and effective. This meta-analysis demonstrated that combined TXA application may be superior to topical or intravenous application of TXA alone. However, larger, high-quality randomized control trials are required for greater confidence in this finding.

## Background

Total hip arthroplasty (THA) is an effective surgical procedure for patients with end-stage hip diseases, but is always accompanied by substantial blood loss and high transfusion rates [1, 2]. Although allogenic blood transfusion may be beneficial to patients, it may induce considerable side effects, such as delay of hip functional recovery, adverse reaction to the transfusion, cardiovascular dysfunction, disease transmission, and joint infection [3–6]. Additionally, allogeneic blood transfusions are costly procedures [7]. Lately, the use of TXA has been expanded for use in THA procedures [8].

Tranexamic acid (TXA) is a synthetic amino acid analogue that acts as a competitive inhibitor of plasminogen and finally interferes with fibrinolysis [9]. TXA can decrease blood loss and reduce transfusion requirements, and has been successfully used in some surgical procedures including THA [8, 10–12]. Intravenous or topical TXA administration for total hip arthroplasty has been demonstrated to be effective and safe in total hip arthroplasty [8, 13–16]. However, which of these two administration routes may be more effective remains controversial due to different merits and drawbacks. The combined administration of TXA has been used in total knee arthroplasty with showed satisfactory results [17–19]. Therefore, we proposed a hypothesis that the combined application of TXA in THA may be the most effective administration strategy without increased rate of thrombotic events.

The efficacy and safety of combined application versus topical or intravenous application of tranexamic acid in THA remains controversial. To our knowledge, no meta-analysis had been previously reported. Therefore, we conducted this meta-analysis to compare these treatment strategies, evaluating outcomes of total blood loss, postoperative hemoglobin decline, transfusion rates, the incidence rate of deep vein thrombosis (DVT), and the incidence rate of pulmonary embolisms (PE).

## Methods

### Search strategy

**Fig. 1:**
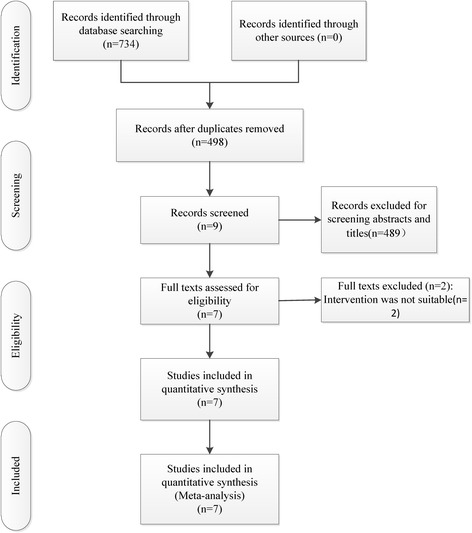
The flow chart of studies selecting

We searched the electronic databases including PubMed, EMBASE, the Cochrane Library, Web of Science, the Chinese Biomedical Literature database, the CNKI database, and Wanfang Data until September 2016. The references of the included literatures were also checked for potentially relevant studies. There were no language restrictions. The data of the included studies were analyzed using RevMan 5.3 software. The key words used in search methods including “tranexamic acid”, “total hip arthroplasty”. We made a joint retrieval of free and subject words. The Boolean operators were used to combine them. The search results were showed in Fig. 1.

### Selection criteria

Trials were eligible for inclusion if they met the following criteria: 1) Randomized controlled trials or retrospective control trials; 2) The study compared the efficacy and safety of combined application versus topical or intravenous application of tranexamic acid in primary THA; 3) The study evaluated at least one of the outcome measures of total blood loss, postoperative hemoglobin decline, transfusion rates, deep vein thrombosis (DVT) incidence, and pulmonary embolism (PE) incidence. Studies were excluded if: 1) Patients received other hemostasis treatment; 2) Patients had history of thrombotic events (PE or DVT).

### Data extraction

The included studies were examined by two investigators and key data were extracted including first author’s name, the published year, study type, sample size, mean age, anesthesia methods, TXA intervention, prosthesis type, prophylactic antithrombotic therapy, transfusion trigger, and surgical outcomes for the meta-analysis (including total blood loss, postoperative hemoglobin decline, transfusion rates, and postoperative complications: VT, PE). When disagreement existed, it was resolved by consulting another investigator.

### Quality assessment

The quality of the randomized controlled trials was assessed according to the Cochrane risk assessment scale and included details of the methods of random sequence generation, allocation concealment, blinding, incomplete outcome data, selective outcome reporting, and other sources of bias. The Methodological Index for Non-Randomized Studies (MINORS) was used to assess non-RCTs. The assessments were performed by two investigators independently. Any disagreement was resolved by a third reviewer.

### Statistical analysis

This meta-analysis was performed using RevMan 5.3. The heterogeneity level among included studies was assessed by using the value of P and *I* ^2^. If *I* ^2^ < 50%, *P* > 0.1, this represented low heterogeneity between studies, and a fixed-effect model was used, otherwise, a random-effect model was used. For continuous outcomes, we calculated the mean difference (MD) with 95% confidence interval (CI). The risk difference (RD) with 95% CI was calculated for dichotomous data. If necessary, sensitivity analysis was conducted to identify the origins of the significant heterogeneity.

## Results

### Description of studies

The search of electronic database identified 498 potentially relevant references for preliminary review. After the scan of titles and abstracts, 489 studies were excluded. After the full texts were assessed for eligibility, six RCTs and a retrospective study published between 2014 and 2016 were selected for inclusion [20–26]. The characteristics of the included studies are shown in Table 1.

**Table 1:** The characteristics of included studies

Study (year)	Number	Mean age	Anesthesia methods	TXA intervention	Prosthesis type	Thromboprophylaxis	Transfusion criteria
Xie 2016 [26]	70/70/70(T/I/C)	62.2/59.5/60.5(T/I/C)	General anesthesia	T:3 g; I: 1.5 g; C:1 g (IV) + 2 g (T)	Cementless	Enoxaparin + Physical therapy	Hb <7 g/dL
Yue 2015 [21]	136/238/357(T/I/C)	60.3/62.6/61.9(T/I/C)	–	T:3 g; I:15 mg/kg; TXA; C:15 mg/kg(IV) + 1.5 g (T)	Cementless	Low-molecular-weight heparin + Physical therapy	Hb <7 g/dL
Zhang 2015 [20]	34/34/34(T/I/C)	65.2/63.4/64.7(T/I/C)	–	T:0.1 g; I: l g; C:1 g(IV) + 0.1 g(T)	Cemented	Enoxaparin + rivaroxaban	Hb <7 g/dL
Zhao 2015 [25]	44/48/44(T/I/C)	62.2/59.8/57.6(T/I/C)	Continuous epidural anesthesia	T:1 g; I:1 g; C:1 g(IV) + 1 g(T)	Cementless	Rivaroxaban + Physical therapy	Hb < 8 g/dL
Lu 2016 [22]	141/141/141(T/I/C)	66.8/66.0/65.0(T/I/C)	General anesthesia	T: 2 g; I: 30 mg/kg TXA; C:30 mg/kg (IV) + 2 g (T)	Cementless	–	Hb <7 g/dL
Zhu 2016 [23]	20/20/20(T/I/C)	58.0/60.4/62.0(T/I/C)	–	T: 2 g; I: 15 mg/kg TXA; C:15 mg/kg (IV) + 2 g (T)	Cementless	–	–
Zeng 2016 [24]	50/50(/I/C)	54.0/53.6(/I/C)	–	I:15 mg/kg TXA; C:15 mg/kg (IV) + 1 g (T)	–	Low-molecular-weight heparin + Physical therapy	Hb <7 g/dL

*C* combined group, *T* topical group, *I* intravenous group, *IV* intravenous injection, *Hb* hemoglobin

The selected studies reported comparable baseline characteristics of treatment groups regarding age, gender, Body Mass Index (BMI), and preoperative laboratory parameters (red blood cell specific volume: Hct and hemoglobin: Hb). Six studies reported the transfusion trigger: less than 70 g/L for five studies and 80 g/L for the remaining study. For thromboprophylaxis, two studies used low molecular weight heparin (LMWH) combined with physical therapy, one used enoxaparin combined with physical therapy, one used enoxaparin and rivaroxaban, and one used rivaroxaban combined with physical therapy. Two studies did not report the use of thromboprophylaxis.

### Risk of bias assessment

**Fig. 2:**
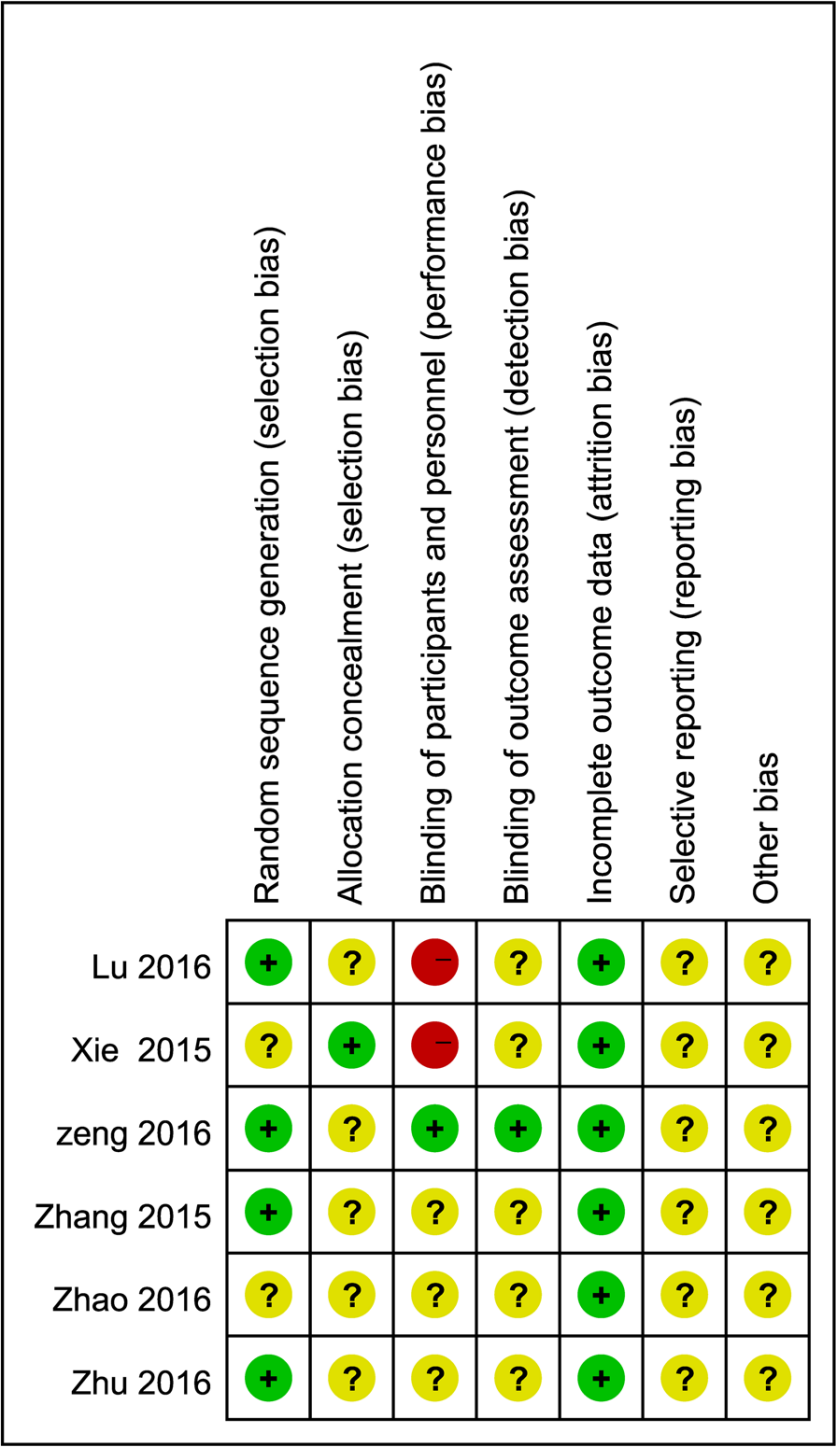
The quality of the randomized controlled trials

**Fig. 3:**
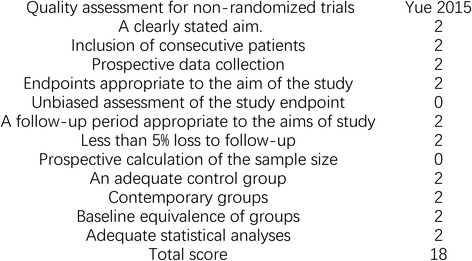
Quality assessment for the non-randomized trial

All included RCTs showed clear inclusion and exclusion criteria. In four [20, 22–24] of the included RCTs, the randomization algorithm was generated from a blinded biostatistician or a computer. In one RCT [26], the allocation concealment was performed using opaque sealed envelopes. One RCT [24] provided details of the double blinding for treatments and the blinding of outcome assessment. All RCTs reported complete outcome data. The results of the quality of the included RCTs are shown in Fig. 2. The MINORS scale was used to assess the retrospective study, as presented in Fig. 3.

### Outcomes measures

#### Total blood loss (ml)

1. Comparisons between the combined group and the topical group.
  Six studies (1111patients) [20–23, 25, 26] investigated total blood loss. Heterogeneity was significant in the studies (*P* <0.00001; *I* ^2^ = 95%), so the random-effects model was used. The pooled result revealed that combined application of TXA significantly reduced total blood loss compared to topical application of TXA. (MD = −162, 95% CI: −265 to −60, *P* = 0.002. Figure 4a).

**Fig. 4:**
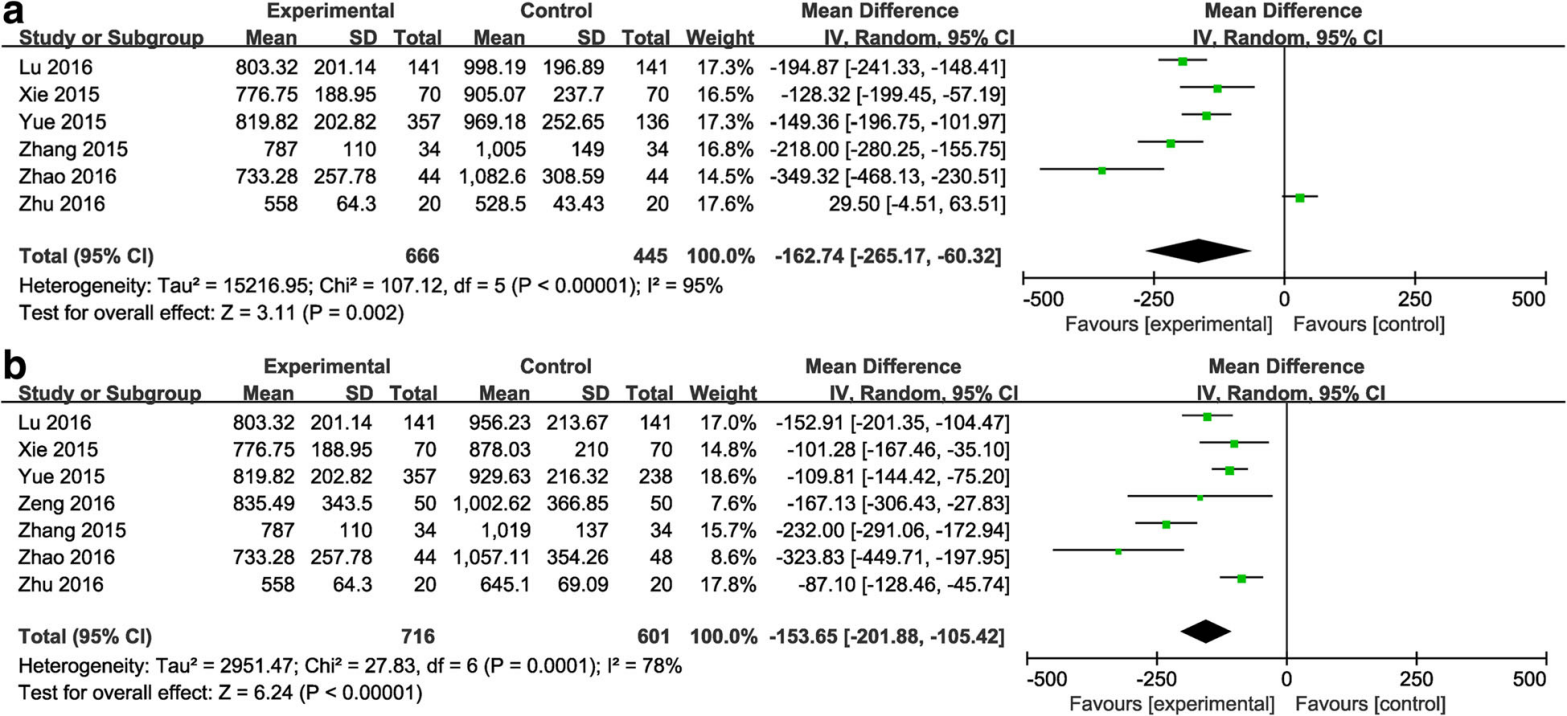
**a**: The comparison between the combined group and the topical group in total blood loss. **b**: The comparison between the combined group and the intravenous group in total blood loss
2. Comparisons between the combined group and the intravenous group.
  Seven studies (for a total of 1317 patients) [20–26] compared total blood loss. Heterogeneity was significant in the studies (*P* =0.0001; *I* ^2^ = 78%), so the random-effects model was used. The pooled result revealed that the combined group exhibited lower total blood loss than the intravenous group (MD = −153, 95% CI: −201 to −105, *P* <0.00001. Figure 4b).

#### Postoperative hemoglobin decline (g/dl)

1. Comparison between the combined group and the topical group.
  Four reports (including 1003 patients) [21, 22, 25, 26] reported the outcome of postoperative hemoglobin decline. Significant heterogeneity was detected in the studies (*P* =0.007; *I* ^2^ = 75%), so the random-effects model was used. The pooled results showed that the combined application group showed postoperative hemoglobin decline (MD = −1.06, 95% CI: −1.30 to −0.82, *P* < 0.00001; Fig. 5a).

**Fig. 5:**
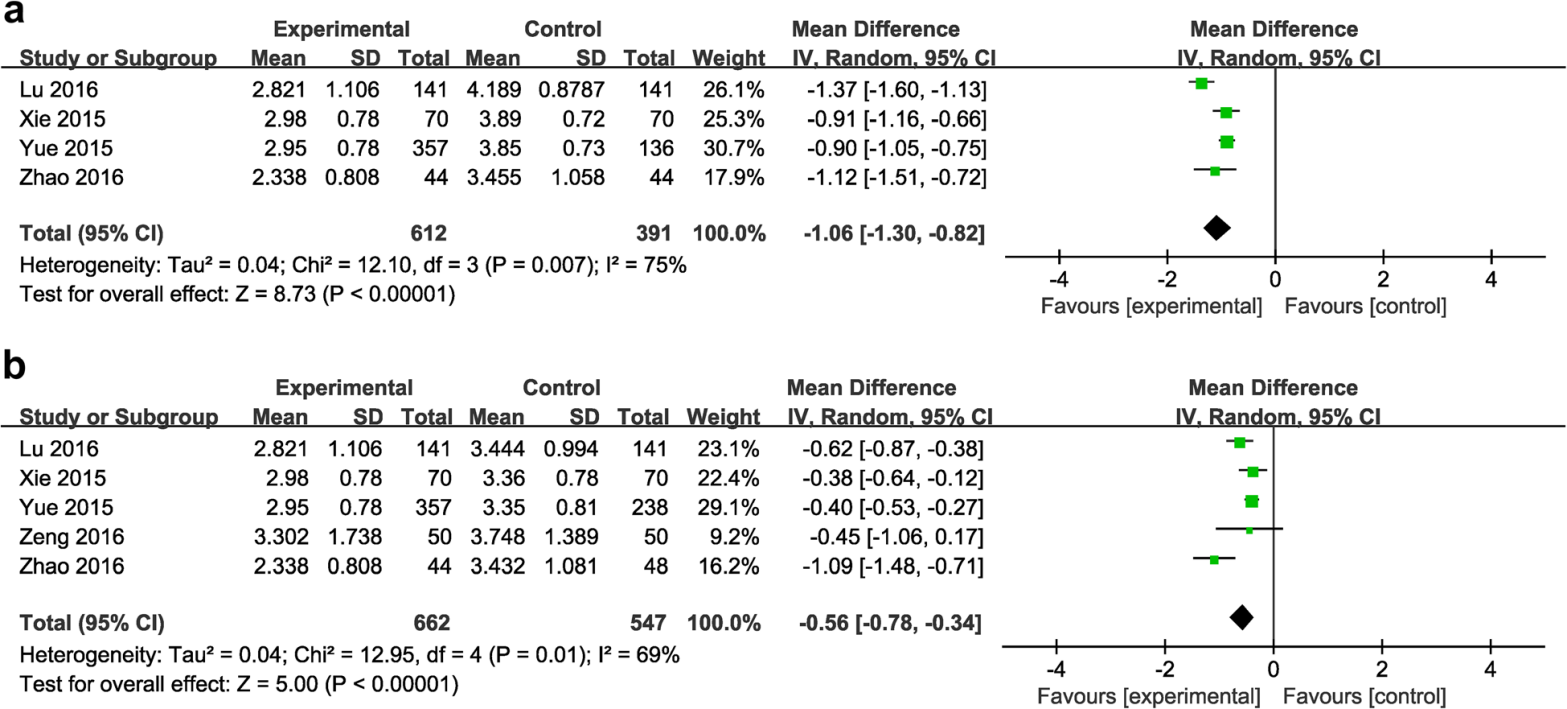
**a**: The comparison between the combined group and the topical group in postoperative hemoglobin decline. **b**: The comparison between the combined group and the intravenous group in postoperative hemoglobin decline
2. Comparison between the combined group and the intravenous group.
  Five studies (including 1209 patients) [21, 22, 24–26] reported the outcome of postoperative hemoglobin decline. Heterogeneity was significant in the studies (*P* =0.01; *I* ^2^ = 69%); so, the random-effects model was used. The pooled results showed that the combined application group exhibited lower postoperative hemoglobin decline (MD = −0.56, 95% CI: −0.78 to −0.34, *P* < 0.00001; Fig. 5b).

#### Transfusion rates

1. Comparison between the combined group and the topical group.
  Five studies (including 1071 patients) [20–22, 25, 26] compared transfusion rates. No significant heterogeneity was detected in the studies (*P* =0.97; *I* ^2^ = 0%). Therefore, the fixed-effects model was used for analysis. The results showed the combined application group had lower transfusion rates (RD = −0.05, 95% CI: −0.08 to −0.02, *P* = 0.0005; Fig. 6a).

**Fig. 6:**
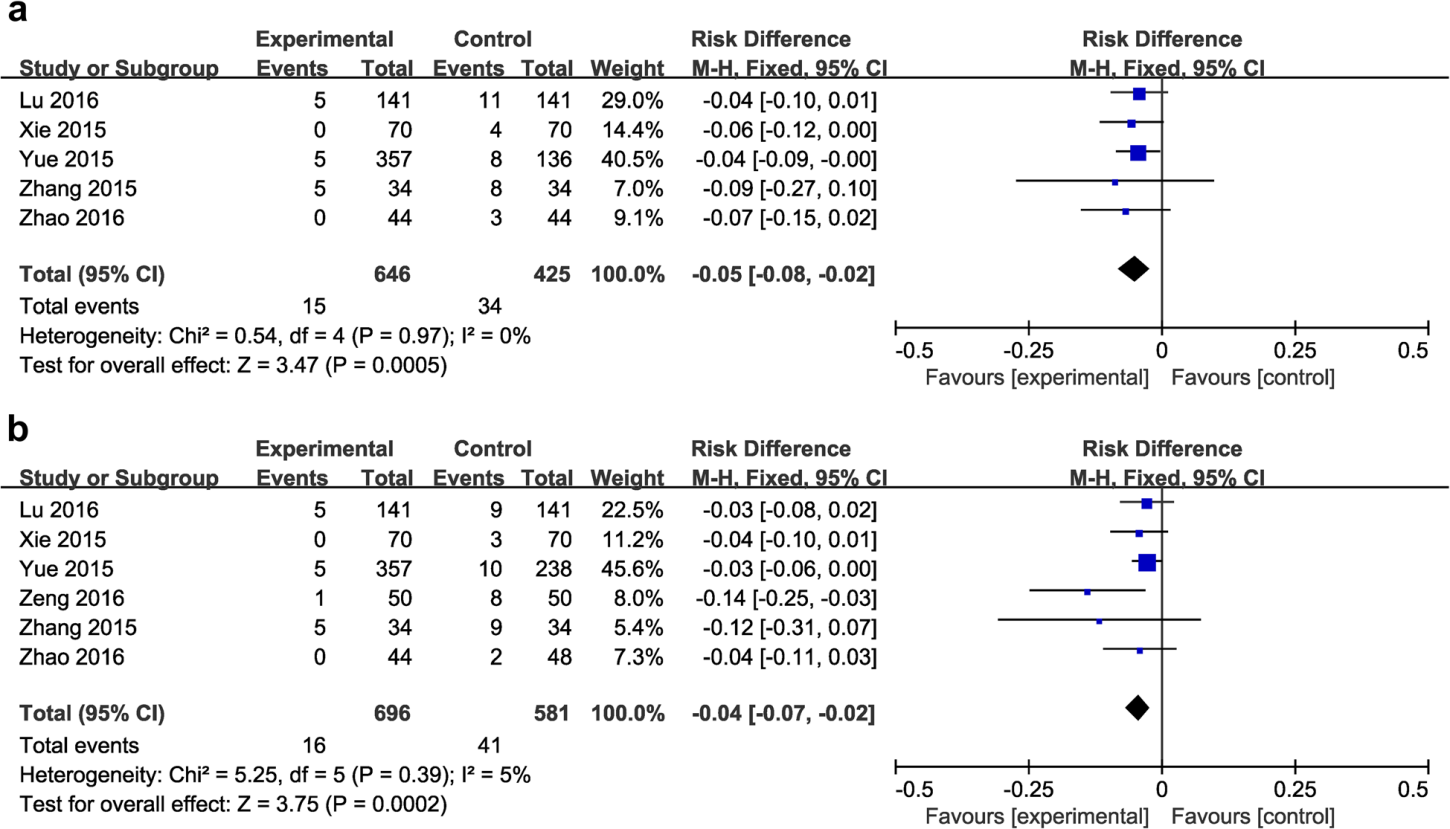
**a**: The comparison between the combined group and the topical group in transfusion rates. **b**: The comparison between the combined group and the intravenous group in transfusion rates
2. Comparison between the combined group and the intravenous group.
  Six studies (including 1277 patients) [20–22, 24–26] compared transfusion rates. No significant heterogeneity was detected in the studies (*P* =0.39; *I* ^2^ = 5%). Therefore, the fixed- effects model was used for analysis. The results showed that the combined application group had lower transfusion rates (RD = −0.04, 95% CI: −0.07 to −0.02, *P* = 0.0002; Fig. 6b).

#### Deep vein thrombosis (DVT)

1. Comparison between the combined group and the topical group.
  Six articles (for a total of 1111 patients) [20–23, 25, 26] reported the incidence of DVT. No significant heterogeneity was found (*P* = 0.87; *I* ^2^ = 0%), so the fixed-effects model was used. It showed no significant difference between the groups (RD = 0.00, 95% CI: −0.01 to 0.02, *P* = 0.93; Fig. 7a).

**Fig. 7:**
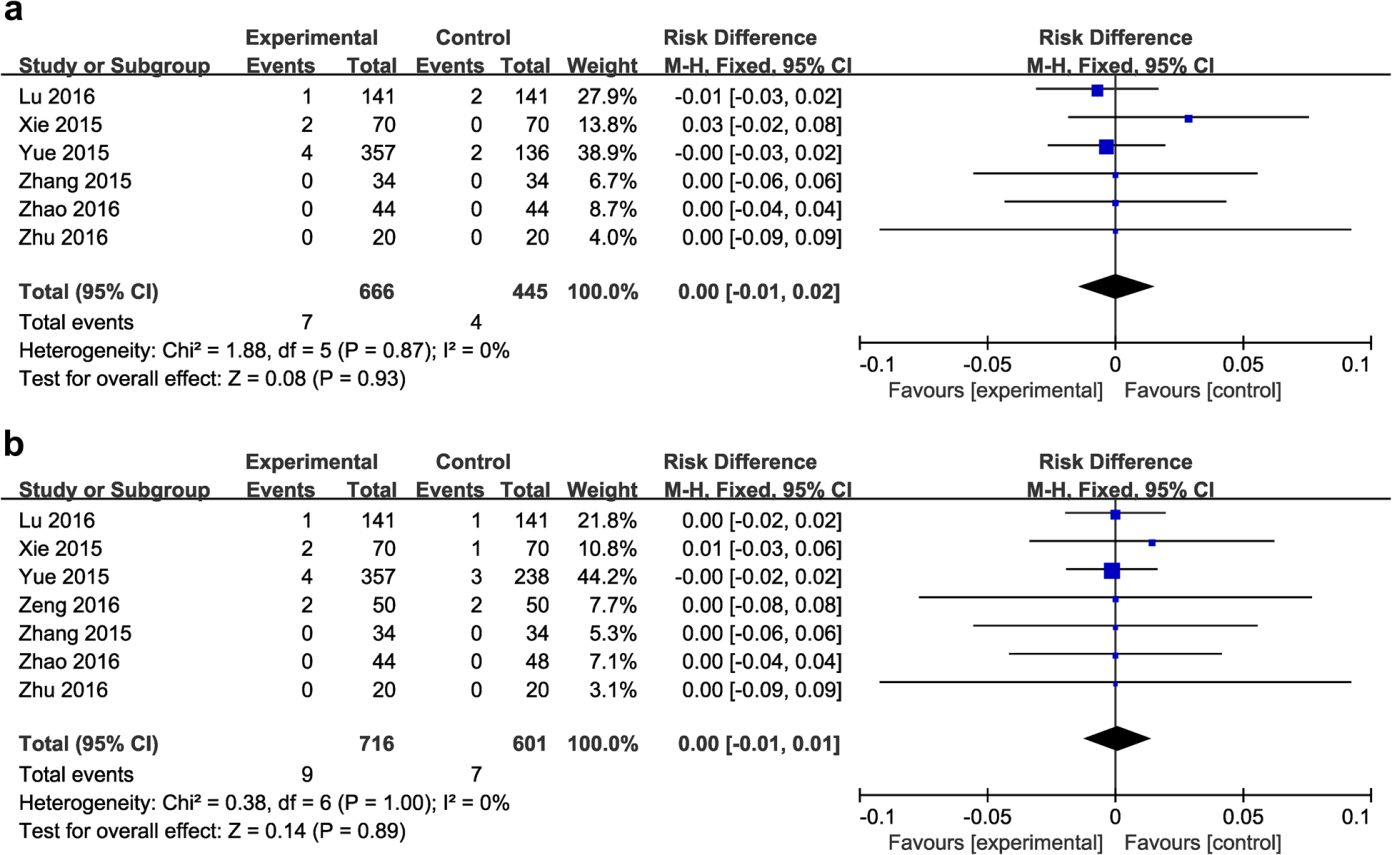
**a**: The comparison between the combined group and the topical group in the incidence rate of DVT. **b**: The comparison between the combined group and the intravenous group in the incidence rate of DVT
2. Comparison between the combined group and the intravenous group.
  Seven articles (for a total of 1317 patients) [20–26] reported the incidence of DVT. No significant heterogeneity was found (*P* =1; *I* ^2^ = 0%), so the fixed-effects model was used. It showed no significant difference between the groups (RD = 0.00, 95% CI: −0.01 to 0.01, *P* = 0.89; Fig. 7b).

#### Pulmonary embolism (PE)

1. Comparison between the combined group and the topical group.
  PE was reported in four included studies (1003 patients) [21, 22, 25, 26]. No significant heterogeneity was found (*P* = 1; *I* ^2^ = 0%), therefore, the fixed-effects model was used. It manifested no significant difference between the treatments (RD = 0.00, 95% CI: −0.01 to 0.01, *P* = 1; Fig. 8a).

**Fig. 8:**
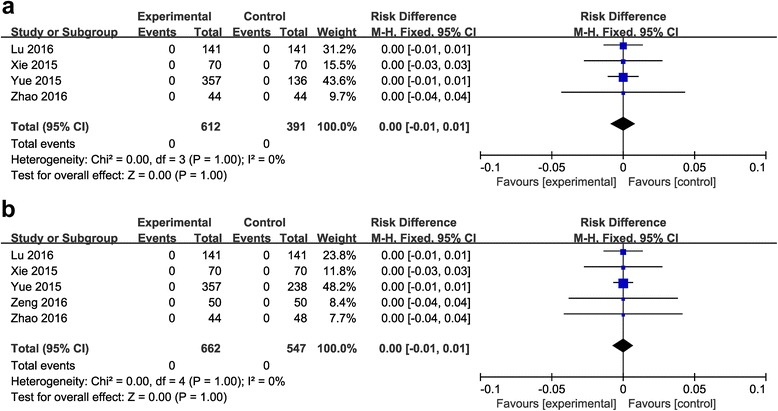
**a**: The comparison between the combined group and the topical group in the incidence rate of PE. **b**: The comparison between the combined group and the intravenous group in the incidence rate of PE
2. Comparison between the combined group and the intravenous group.
  PE was reported in five included studies (1209 patients) [21, 22, 24–26]. No significant heterogeneity was found (*P* = 1; *I* ^2^ = 0%), therefore, the fixed-effects model was used. It manifested no significant difference between the groups (RD = 0.00, 95% CI: −0.01 to 0.01, *P* = 1; Fig. 8b).

## Discussion

To our knowledge, this is the first meta-analysis comparing the hemostatic efficacy and safety of the combined application of intravenous and topical application of tranexamic acid in primary THA. The most important finding of our meta-analysis was that the combined application of TXA was correlated with a significant lower total blood loss and transfusion requirements without increasing rate of thrombotic events (DVT or PE).

TXA has been shown to decrease blood loss and reduce transfusion requirements, and has been successfully used in surgical procedures including THA [8, 10–12]. Intravenous or topical TXA use alone has been demonstrated to be effective and safe in total hip arthroplasty [8, 13–16]. When TXA is given intravenously, it is widely distributed throughout the extracellular and intracellular compartment, and rapidly reaches a maximum plasma concentration (in 5 to 15 min) [26, 27]. Local fibrinolysis can be inhibited from the start of surgery [26]. Topical application of TXA may maintain a maximum local level for hemostasis by maintaining fibrin clotting [26, 28]. The combined application of TXA has been used in total knee arthroplasty with satisfactory results [17–19]. To our knowledge, no meta-analysis has evaluated the efficacy and safety of combined application versus topical or intravenous administration of TXA in THA. The primary endpoints of our study included total blood loss, postoperative hemoglobin decline, and transfusion rates. Our results revealed that the combined group had lower total blood loss. Xie et al. [26] reported that compared to IV TXA or topical TXA, combined administration of TXA can additionally reduce total blood loss by 12 and 14%, respectively Zeng et al. [24] also reported that the combined group had significantly lower total blood loss than the intravenous group. The research results of Zhang et al. [20] and Zhao et al. [25] are consistent with our findings. Yue et al. [21] also favored a combined approach by retrospective study.

The result of our meta-analysis evaluating transfusion rates favored the combined group. Zhang et al. [20] reported that there was no statistically significant difference in transfusion rates for IV, topical, or combined administration of TXA. All the results of the remaining studies were consistent with the findings of our meta-analysis that the combined group had a lower transfusion rate.

Thrombotic complications could induce severe results and even death after THA, so it is urgent to determine whether combined application of TXA increases the rate of thrombotic events (DVT and PE). Many studies reported that topical or intravenous administration of TXA was not associated with an increased rate of thrombotic events [29–32]. In our meta-analysis, all the included studies reported no significant difference in the incidence rate of DVT or PE in the combined, topical, or intravenous treatment groups. This finding was in accordance with the results of our meta-analysis. Sensitivity analysis based on the study type also showed no statistical difference. Although the methods of thromboprophylaxis differed in the included studies, our study did not show any significant heterogeneity in DVT and PE among these studies. However, due to the small sample size and short follow-up in most of the included studies, larger studies with more patients and longer follow-up are required to confirm whether the combined treatment strategy is safe without increasing thrombotic events.

Significant heterogeneity was detected in some outcomes in our meta-analysis. The sensitivity analysis based on study type demonstrated no statistically significant difference in the outcomes. In four of the included RCTs, a blinded biostatistician or a computer generated the randomization algorithm. In one RCT,the allocation concealment was performed using opaque sealed envelopes. One RCT included details of the double blinding and the blinding of the outcome assessment.

There are several limits of this meta-analysis. We included a retrospective study in our analysis due to the limited amount of published RCTs. This inclusion decreased the evidence level of the meta-analysis to some extent. Although we searched electronic databases systematically, some relevant studies might have been missed due to publication bias. Additionally, the sample size was very small in most of the included studies.

In addition, there was clinical heterogeneity in the reports as follows: 1) Differences in surgical time, technique, approaches, and postoperative measures. 2) Different anesthesia methods, which can affect blood loss and transfusion requirements (general anesthesia was related with an increased rate of adverse events and blood transfusions compared with spinal anesthesia) [33, 34]. However, a larger sample size of patients and multi-center studies are required to confirm this conclusion. 3) Differences in the TXA dose, especially Zhang 2015 [20], there was insufficient data to perform subgroup analysis and the optimal dose still remains controversial. In order to clarify the contributions of all these identified issues, additional studies with larger sample sizes are needed.

The limitations of this study were as follows: 1) The relatively small sample size of each primary study, especially Zhu 2016 [23]; and there was significant heterogeneity in total blood loss and postoperative hemoglobin decline. However, we could not conduct subgroup analysis due to insufficient data.2)In some RCTs, the methods of random sequence generation, allocation concealment, blinding, and selective outcome reporting were unclear or not described, which may influence the stability of our outcomes to some extent. 3)A retrospective study was included due to the limitation of studies, which also lowered the robust analysis. 4) Differences in surgical time, technique, approaches, and postoperative measures may have influenced the results. 5) There is publication bias.

## Conclusion

The group that received the combined treatment had lower total blood loss, postoperative hemoglobin decline, and transfusion rates without an increased rate of thrombotic events (DVT or PE). The topical or intravenous use of TXA in primary THA is generally considered to be safe and effective. This meta-analysis demonstrated that combined TXA application may be superior to topical or intravenous application of TXA alone. However, larger, high-quality randomized control trials are required for greater confidence in this finding.

## Abbreviations

BMI: Body mass index
DVT: Deep vein thrombosis
Hb: Hemoglobin
Hct: Red blood cell specific volume
IV: Intravenous
PE: Pulmonary embolisms
THA: Total hip arthroplasty
TXA: Tranexamic acid
